# Non-caveolar caveolin-1 expression in prostate cancer cells promotes lymphangiogenesis

**DOI:** 10.18632/oncoscience.180

**Published:** 2015-08-03

**Authors:** Zeyad D. Nassar, Michelle M. Hill, Robert G. Parton, Mathias Francois, Marie-Odile Parat

**Affiliations:** ^1^ The University of Queensland, School of Pharmacy, QLD, Australia; ^2^ The University of Queensland Diamantina Institute, The University of Queensland, Translational Research Institute, QLD, Australia; ^3^ The University of Queensland, Institute for Molecular Bioscience, QLD, Australia

**Keywords:** caveolae, prostate cancer, lymphatic endothelial cells, lymphangiogenesis, VEGF

## Abstract

Lymphangiogenesis allows prostate cancer (PCa) lymphatic metastasis, which is associated with poor prognosis and short survival rates. Caveolin-1 (Cav-1) is a membrane protein localized in caveolae, but also exists in non-caveolar, cellular or extracellular forms. Cav-1 is overexpressed in PCa, promotes prostate tumour progression and metastasis. We investigated the effect of caveolar and non-caveolar Cav-1 on PCa lymphangiogenic potential. Cav-1 was down-regulated in PC3 and DU145, and ectopically expressed in LNCaP cells. The effect of PCa cell conditioned media on lymphatic endothelial cell (LEC) viability, chemotaxis, chemokinesis and differentiation was assessed. The effect of Cav-1 on PCa cell expression of lymphangiogenesis-modulators VEGF-A and VEGF-C was assessed using qPCR and ELISA of the conditioned medium. Non-caveolar Cav-1, whether exogenous or endogenous (in LNCaP and PC3 cells, respectively) enhanced LEC proliferation, migration and differentiation. In contrast, caveolar Cav-1 (in DU145 cells) did not significantly affect PCa cell lymphangiogenic potential. The effect of non-caveolar Cav-1 on LECs was mediated by increased expression of VEGF-A as demonstrated by neutralization by anti-VEGF-A antibody. This study unveils for the first time a crucial role for non-caveolar Cav-1 in modulating PCa cell expression of VEGF-A and subsequent LEC proliferation, migration and tube formation.

## INTRODUCTION

Lymph node (LN) metastasis significantly affects prostate cancer prognosis [1]. Lymphangiogenesis, the formation of new lymphatic vessels from pre-established ones, is one of the mechanisms that promote prostate cancer LN metastasis. A positive association between lymphangiogenic factors such as VEGF-A, -C, and -D, and lymph node metastasis, pathological grade, and poor prognosis of the disease is documented [2–6]. Understanding the biology of PCa lymphangiogenesis, and developing therapeutic strategies that interfere with this process, is an active field of research [7]. Together with angiogenesis, lymphangiogenesis is an effective therapeutic target in PCa [8–10].

Caveolae are small plasma membrane pits (60–80 nm) with important functions in cellular processes such as signalling, endocytosis, migration, adhesion and trafficking. Two proteins are essential in caveola formation, namely caveolin-1 (Cav-1) and PTRF [11]. Both tumour suppressive and tumour-promoting roles are reported for Cav-1 in cancer, depending on the cancer type, the tumour stage, and the cellular compartment that is analysed [11–15]. In prostate cancer however, overwhelming evidence implicates Cav-1 as being associated with, and mediating, increased aggressiveness; Cav-1 overexpression in prostate cancer clinical samples correlates with disease stage [16], grade [17], metastasis [16], androgen insensitivity [18] angiogenesis [19] and poor outcome [20]. *In vitro* and preclinical studies further support that Cav-1 expression in prostate cancer cells increases tumour growth, invasion, angiogenesis and ultimately metastasis [18, 21-27]. Cav-1 can be secreted by prostate cancer cells and displays paracrine and endocrine functions [18, 23, 24, 28]. Cav-1 is found in the circulation of PCa patients and has been proposed as a diagnostic, prognostic, or therapeutic efficacy marker [28, 29].

Systemic administration of anti-Cav-1 antibody for three weeks to mice orthotopically injected with Cav-1-secreting PCa cells decreases cancer cell volume in lymph nodes [18]. However, there is no published study testing whether manipulating Cav-1 expression in PCa cells modulates lymphangiogenesis. Moreover, most of the work identifying the effect of Cav-1 expression on PCa aggressiveness and angiogenic potential [18, 21, 23, 30, 31] precedes the recognition that in the absence of PTRF, cells cannot form caveolae and Cav-1 resides in a different compartment [32] and therefore did not differentiate between the effects of caveolar Cav-1 and non-caveolar Cav-1, which we are now able to dissect out [11, 33-35]. In the current study, we investigated the effect of caveolar and non-caveolar Cav-1 in three PCa cell lines on their lymphangiogenic phenotype, and unveiled a mechanism of Cav-1 pro-lymphangiogenic action in PCa.

## RESULTS

### Modulation of Cav-1 expression

**Figure 1:**
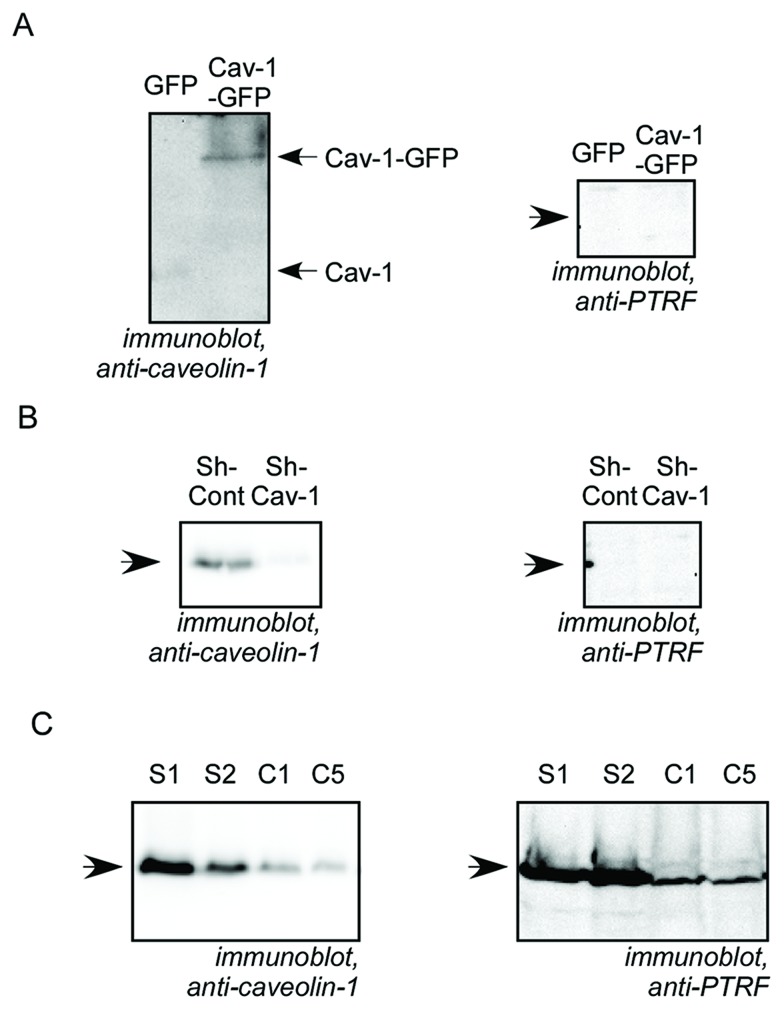
Characterization of Cav-1 and PTRF expression in prostate cancer cell lines Cell lysates from prostate cancer cells were separated by SDS-PAGE and analysed by immunobloting with anti-caveolin-1 or anti-PTRF antibody as indicated. **(A)** LNCaP cells stably expressing GFP or Cav-1-GFP. **(B)** PC3 pooled cells stably transfected with non-targeting, control shRNA (sh-Cont) or with Cav-1 shRNA (sh-Cav-1). **(C)** DU145 cell clones stably transfected with scrambled shRNA (S1 and S2 clones) or with Cav-1 shRNA (C1 and C5 clones).

In order to study the role of Cav-1 expression by PCa cells on lymphangiogenesis, we employed three PCa cell models in which Cav-1 expression was experimentally manipulated: Cav-1-GFP was exogenously expressed in androgen-sensitive human LNCaP cells, which do not express endogenous Cav-1. Cav-1 was stably down-regulated via shRNA in androgen-independent PC3 and DU145 cells, which express abundant endogenous Cav-1. Alteration of Cav-1 expression in the three cell lines was confirmed by Western blot analysis (Figure 1 and S1). The cell lines differ in their expression of the cytoplasmic protein PTRF, which is necessary for caveola formation, thus allowing each combination of Cav-1 expression and caveola formation, as summarized in Table 1. Cav-1 down-regulation in DU145 resulted in reduced expression of PTRF. Cav-1 down-regulation had no effect on PTRF expression in PC3 cells which lack PTRF. Cav-1 expression in LNCaP cells, which also lack PTRF, did not restore PTRF expression. It is important to note that in Cav-1-GFP LNCaP and in sh-Cont PC3, Cav-1 exists in a non-caveolar form because neither cell line expresses PTRF, whereas in S1 and S2 DU145, Cav-1 is able to form caveolae [34] since the cells express PTRF (Table 1).

**Table 1:** Characteristics of the PCa cells with experimentally altered Cav-1 expression used in this study

Transfection	LNCaP		PC3		DU145	
	GFP	Cav-1-GFP	Sh-Cont	Sh-Cav-1	S1, S2	C1, C5
Caveolin-1	−	+	+	↘↘	+	↘↘
PTRF	−	−	−	−	+	↘
Caveolae	−	−	−	−	+	↘↘
		Non-caveolarectopic Cav-1	Non-caveolarendogenous Cav-1		Caveolarendogenous Cav-1	

### Effect of PCa Cav-1 on lymphatic endothelial cell (LEC) proliferation

**Figure 2:**
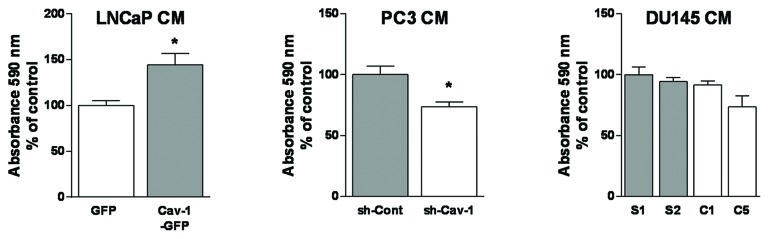
Effect of Cav-1 expression in prostate cancer cells on secretome-modulated LEC viability LEC viability after 48 h exposure to prostate cancer cell-conditioned media was assessed using the MTT assay. Results are reported as percent of LEC viability compared to the control PCa cell conditioned medium and shown as mean ± S.E.M. (n=3 separate experiments), *p<0.05.

The effect of prostate cancer cell-conditioned medium on LEC proliferation was evaluated by the MTT assay. Ectopic expression of Cav-1 in LNCaP cells enhanced LEC proliferation significantly compared to cells exposed to the CM of control cells. Similarly, LECs exposed to the conditioned medium of Cav-1-down-regulated PC3 cells showed significantly less proliferation than LECs treated with the CM of control cells expressing endogenous Cav-1. In contrast, down-regulation of Cav-1 expression in DU145 cells only marginally lowered LEC viability, and the change was not statistically significant (Figure 2). These results indicate that Cav-1 expression in LNCaP and PC3, but not in DU145, promotes LEC proliferation.

### Effect of PCa Cav-1 on LEC migration

**Figure 3:**
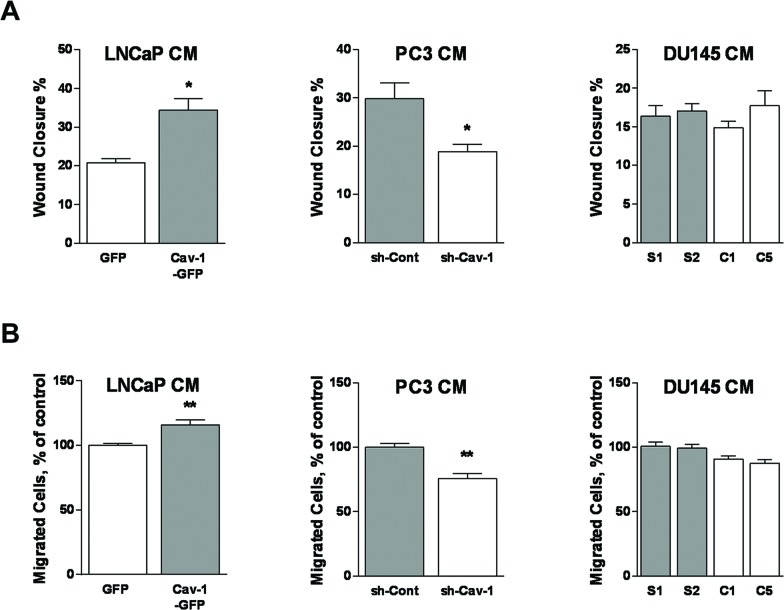
Effect of Cav-1 expression in prostate cancer cells on LEC chemotaxis and chemokinesis **(A)** LEC ability to migrate randomly in two-dimension was tested using a wound healing assay during which cells were incubated in various prostate cancer cell-conditioned media for 6 h. Results are reported as percent of wound closure and shown as mean ± S.E.M. (n=3 separate experiments), *p<0.05. **(B)** LEC directional migration toward conditioned media from each prostate cancer cell line was assessed using the modified Boyden chamber assay. Results are reported as percent of the migration to conditioned medium of PCa control cells and shown as mean ± S.E.M. (n=3-5 separate experiments), **p<0.01.

We tested the ability of PCa cell-conditioned medium to promote chemokinesis by performing a scratch wound migration assay. LECs exposed to the conditioned medium of LNCaP cells expressing Cav-1 migrated significantly more than LECs exposed to CM of cells lacking Cav-1 (Figure 3a). In addition, the attenuation of Cav-1 expression in PC3 cells led to significantly decreased LEC migration, while there was no significant difference in the random migration rate between LECs exposed to conditioned medium of control DU145 versus Cav-1-down-regulated DU145 cells (Figure 3a). To evaluate the impact of Cav-1 expression in PCa cells on LEC chemotaxis, a modified Boyden chamber migration assay was conducted using PCa cell-conditioned media in the lower wells. The number of migrated LECs was significantly higher when using the medium of Cav-1-expressing LNCaP cells than when using the medium of LNCaP devoid of Cav-1. Similarly, knocking-down Cav-1 expression in PC3 decreased the production of LEC-attracting factor(s) compared with control PC3 cells. However, there was no statistically significant effect of Cav-1 down-regulation in DU145 cells on directed LEC migration (Figure 3b). These results suggest that Cav-1 expression in LNCaP and PC3, but not DU145, promotes LEC migration.

### Effect of Cav-1 on differentiation of LEC into tube-like structures

**Figure 4:**
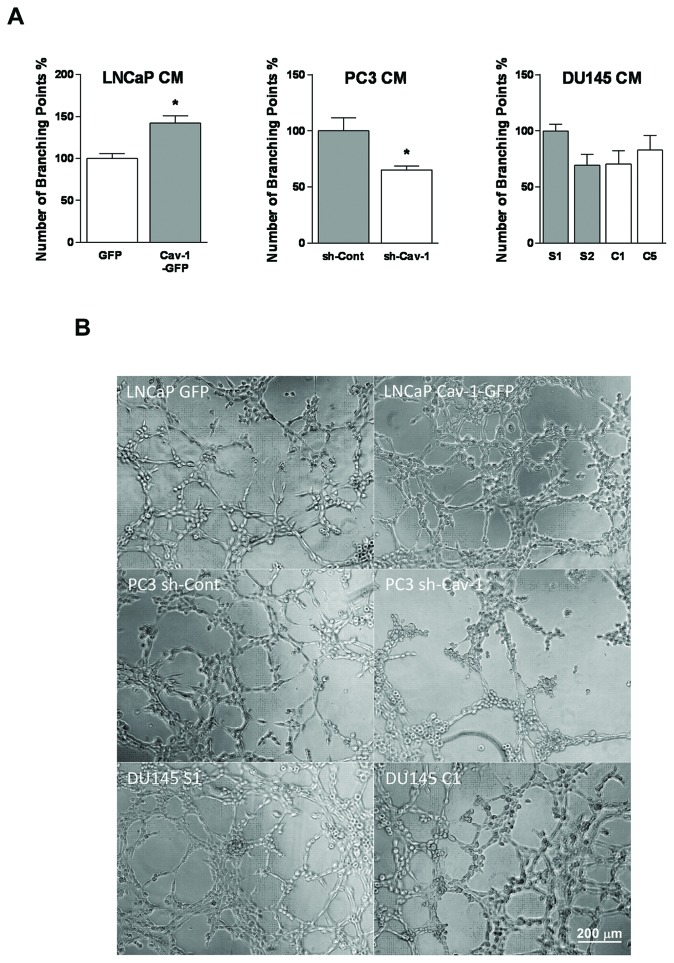
Effect of Cav-1 expression in prostate cancer cells on *in vitro* lymphangiogenesis **(A)** LEC differentiation into tube-like structures was investigated by plating them on Matrigel− -pre-coated 96-well plates and exposing them to various prostate cancer cell-conditioned media for 4-6 h. The number of branching points was quantified. Results are reported as percent of control PCa cell conditioned medium and shown as mean ± S.E.M. (n=3 separate experiments), *p<0.05. **(B)** Representative micrographs of tubes formed by LEC exposed to PCa cell-conditioned media.

The consequences of Cav-1 expression in prostate cancer cells on their lymphangiogenic potential were further studied using LEC tube formation on Matrigel™. LECs plated on Matrigel™ and exposed to conditioned medium of Cav-1-expressing LNCaP or PC3 cells formed significantly more tubes than LECs exposed to conditioned media of cells with lacking or down-regulated Cav-1 expression. In contrast, alteration of Cav-1 expression in DU145 cells did not significantly alter LEC differentiation into tube-like structures (Figure 4). These results show that Cav-1 expression in LNCaP and PC3 but not in DU145 promotes LEC differentiation.

### Effect of PCa Cav-1 expression on VEGF-A and VEGF-C production

**Figure 5:**
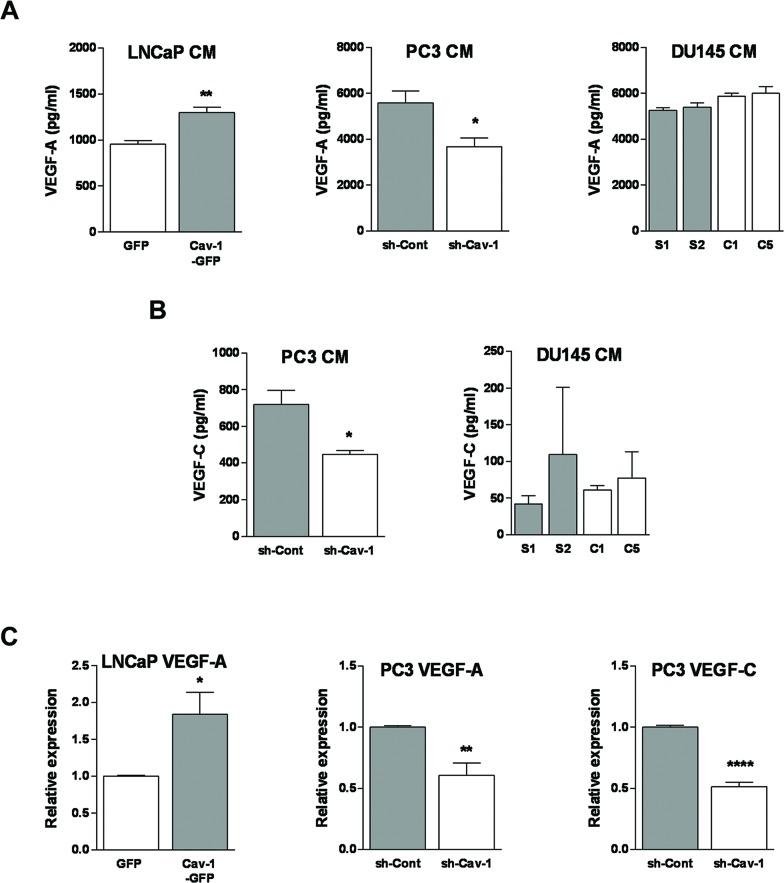
Effect of Cav-1 expression in prostate cancer cells on lymphangiogenesis-promoting growth factors **(A)** Protein level of VEGF-A in prostate cancer cell-conditioned media was measured by ELISA. Results are presented as mean of growth factor concentration ± S.E.M. (n=3 separate experiments), *p<0.05 and **p<0.01. **(B)** Protein level of VEGF-C in prostate cancer cell-conditioned media was measured by ELISA. Results are presented as mean of growth factor concentration ± S.E.M. (n=3 separate experiments), *p<0.05. **(C)** mRNA level of VEGF-A and VEGF-C in prostate cancer cells was determined using real time RT-PCR. Relative expression compared to control cells is shown as mean ± S.E.M. for 6 readings from three independent experiments. *p<0.05, **p<0.01 and ****p<0.0001.

VE GF-A a nd -C a re m a j or re gul a t ors of lymphangiogenesis [36, 37]. In order to unveil the mechanism by which Cav-1 regulates PCa lymphangiogenic potential, we measured the effect of modulating Cav-1 expression on the production of VEGF-A and VEGF-C in PCa cell-conditioned media using ELISA. Overexpression of Cav-1 in LNCaP cells resulted in a significant increase in VEGF-A in the conditioned medium compared with the medium of control cells (Figure 5a). There was no detectable VEGF-C in LNCaP medium whether or not they expressed Cav-1 (not shown). Conditioned media of PC3 with down-regulated Cav-1 expression contained significantly less VEGF-A (Figure 5a) and VEGF-C (Figure 5b) compared with the medium of control PC3 cells. In DU145 cells, reduction of Cav-1 expression did not significant alter VEGF-A or VEGF-C production (Figure 5a, b). These results indicate that Cav-1 promotes expression of VEGF-A in LNCaP and PC3 but not in DU145, and expression of VEGF-C in PC3 but not in DU145.

To determine whether Cav-1 modulated expression of VEGF-A (in PC3 and LNCaP) and VEGF–C (in PC3) at a transcriptional level, we measured the mRNA levels of VEGF-A and VEGF-C using real time RT-qPCR (Figure 5c). The results revealed a significant increase in VEGF-A mRNA in Cav-1-expressing LNCaP cells (*p*=0.0199) and a significant decrease of both VEGF-A (*p*=0.0028) and VEGF-C (*p*<0.0001) mRNAs in Cav-1 down-regulated PC3 cells (Figure 5c). In line with the ELISA results, VEGF-C mRNA was not detected in LNCaP cells (not shown).

### Cav-1 induction of LEC migration is mediated by VEGF-A

**Figure 6:**
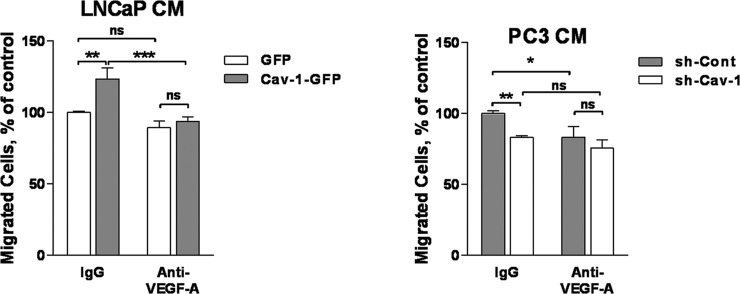
Effect of anti-VEGF-A antibody on prostate cancer cell-conditioned media-induced LEC migration Chemotaxis towards PCa cell-conditioned media was tested using the Boyden chamber assay in the presence or absence of anti-VEGF-A antibody as indicated. Results are reported as percent of the migration to control cell conditioned medium and shown as mean ± S.E.M. (n=3 separate experiment), *p<0.05, **p<0.01, ***p<0.001, ns no statistical significance.

To establish a causality link between the positive effects of Cav-1 expression in prostate cancer on VEGF-A, on the one hand, and functional in vitro assays indicating increased lymphangiogenesis, on the other hand, we tested whether VEGF-A antibody could neutralize the effect of Cav-1 expression on the pro-lymphangiogenic effects of the conditioned media of PCa cells. To that extent, we measured chemotaxis of LEC towards conditioned media from PCa cells added with anti-VEGF-A antibody (for cells that exhibited increased production of VEGF-A, namely LNCaP and PC3). The anti-VEGF-A neutralizing antibody reversed the effect of Cav-1 expression on PCa pro-lymphangiogenic potential: there was no statistically significant difference between sh-Cont and sh-Cav-1 medium or between GFP or Cav-1-GFP LNCaP medium in the presence of anti-VEGF-A (Figure 6), and there was statistically significant differences between IgG- and anti VEGF-A-added conditioned medium of Cav-1-expressing cells (sh-Cont PC3 or Cav-1-GFP LNCaP). These results indicate that VEGF-A at least partly mediates the pro-lymphangiogenic effect of Cav-1 expression in PCa. We did not test whether adding anti-VEGF-C antibody to the conditioned medium would abolish the difference between LEC migration elicited by Cav-1 down-regulated and control PC3 cells, because the concentration found in their conditioned medium (~0.75 ng/ml) is below the minimal concentration of VEGF-C that elicits LEC chemotaxis in our model (10 ng/ml, data not shown). Together with the fact that Cav-1 expression alters the pro-lymphangiogenic potential of LNCaP cells despite their lack of VEGF-C expression, these results suggest that the effect of Cav-1 on PCa lymphangiogenesis in our experiments is likely not VEGF-C mediated.

## DISCUSSION

The role of Cav-1 expression on PCa angiogenesis has been extensively studied. Immunohistochemistry of human prostate tumour specimens revealed that Cav-1 expression is associated with microvessel density and expression of the endothelial cell marker and angiogenesis signalling receptor, VEGF-R2 [19]. Orthotopic prostate tumour xenografts of Cav-1-secreting cells (RM-9) in Cav-1 gene-disrupted mice or their wild type counterparts revealed that the mean tumour weight and microvessel densities in *Cav-1*(+/+) mice were higher than *Cav-1*(−/−) mice [23]. Interestingly, Cav-1 secreted by PCa cells modulates angiogenesis *in vitro* and *in vivo*; in a subcutaneous cancer model where the expression of Cav-1 in LNCaP cells can be induced by tetracyclin, microvessel density, tumour volume, lung metastasis [23] and expression of VEGF-A and TGF-β1 [26] were significantly higher in Cav-1-expressing tumours compared with controls. *In vitro*, recombinant Cav-1 treatment of *Cav-1*(−/−) endothelial cells enhances nitric oxide production, cell migration and tubule formation through stimulation of the PI3K-Akt-eNOS [23], and VEGF/VEGFR2 signalling pathways [31]. Here, we report for the first time the promoting effect of Cav-1 on the ability of prostate cancer cells to elicit lymphangiogenesis.

Lymph nodes are a prime destination for prostate cancer metastatic cells. Three mechanisms have been suggested for prostate cancer lymphatic metastasis: a local extension of cancer cells to surrounding lymphatics (permeation), chemotactic invasion of the cancer cells toward cytokines expressed by the lymphatic vessels, and lymphangiogenesis [38]. To explore the mechanisms that underlie the pro-lymphangiogenic properties of PCa Cav-1, we studied the effect of modulating Cav-1 expression on the production of two master regulators of neovascularization, namely VEGF-A and -C.

In the two cell lines in which Cav-1 expression results in higher ability of the conditioned medium to elicit lymphangiogenesis in the *in vitro* functional assays we used, namely LNCaP and PC3 cells, we show associated changes in VEGF-A production in the conditioned medium. The effect of Cav-1 expression on VEGF-A expression in those cells was further seen at mRNA level. These results are in agreement with a previous study where transient down-regulation of Cav-1 in PC3 reduced the expression of VEGF-A and FGF2, while adenovirus-mediated expression of Cav-1 in LNCaP enhanced the expression of these growth factors [26]. In contrast, we did not detect a significant change in VEGF-A in the conditioned medium of DU145 when Cav-1 expression was altered, and this corroborates the lack of associated outcomes in the functional assays. Importantly, we established a causality link between modulation of VEGF-A expression in PC3 or LNCaP, and promotion of lymphangiogenesis by performing the chemotaxis assay of LEC in the presence of neutralizing anti-VEGF-A antibody.

LNCaP did not express any detectable VEGF-C, either in the conditioned medium or at mRNA level. However in PC3 cells, Cav-1 downregulation was associated with a decrease in VEGF-C transcription and expression. Because the highest concentration of VEGF-C in the conditioned medium of PC3 cells is unable to elicit LEC transmigration in our model, and the anti-VEGF-A completely abolished the difference of migration between conditioned media of Cav-1-expressing and -down-regulated cells, we suggest that the effect of PCa Cav-1 on lymphangiogenesis is mediated by the panangiogenic factor VEGF-A. VEGF-A is best known as a potent angiogenesis stimulator. Although VEGF-A has no role in lymphangiogenesis during embryogenesis, its involvement in tumour lymphangiogenesis and lymphatic metastasis is documented [39–43]. It was reported by Garmy-Susini et al. that both VEGF-A and VEGF-C induce integrin α1β4 expression by lymphatic vessels during lymphangiogenesis [44]. The same group later showed that VEGF-C activation of integrin α1β4 in lymphatic endothelial cells is PI3Kα-mediated [45]. While we have not examined the ability of VEGF-A to activate PI3Kα and α1β4, the ability of VEGF-A to increase phosphorylation of the PI3K downstream signalling molecule Akt, in the same cell line that we have used throughout this study, is documented [46].

Our study employs loss or gain of Cav-1 expression in PC3 and LNCaP cells, respectively, and suggests that in these two cell lines Cav-1 promotes lymphangiogenic potential. However, the down-regulation of Cav-1 in DU145 did not seem to alter their lymphangiogenic potential. These cell lines differ in their expression of PTRF, which is absent in PC3 and LNCaP but abundant in DU145 (Table 1). Both Cav-1 and PTRF are essential for caveola formation [32]. In most cell types and tissues, Cav-1 expression matches PTRF expression, a phenomenon attributed to co-regulation at the transcriptional level and/or to the ability of PTRF to rescue Cav-1 from lysosomal degradation [32, 47]. However, in prostate cancer the balance between the two proteins is lost as indicated by two studies of prostate cancer tissues [35, 48]. In PC3 cells, which recapitulate the abundance of Cav-1 and absence of PTRF seen in clinical samples, caveolae are absent, and non-caveolar Cav-1 can be detected on flat plasma membrane [32]. Distinct pools of Cav-1 therefore need to be taken into account, which can undergo different fates (e.g. lysosomal degradation, inclusion in prostasomes) and play distinct roles, depending on whether or not PTRF is present [11, 32, 49]. Ectopic expression of PTRF in PC3 restores caveola formation and sequestrates Cav-1 in caveolae [32]. It has been suggested that in prostate cancer, the tumour-promoting roles of Cav-1 are due to a non-caveolar form of the protein [11, 33-35]. In the present study, we show that non-caveolar Cav-1 (in PC3 and LNCaP) promotes lymphangiogenesis while caveolar Cav-1 (in DU145) has no effect. We have previously reported that ectopic expression of PTRF in PC3 cells results in an increased expression of Cav-1 [34], and yet reduced PCa cell lymphangiogenic and angiogenic potential, tumour growth and metastasis *in vitro* and *in vivo* [34, 35]. The effect of PTRF on lymphangiogenesis was seen whether or not the cell expressed Cav-1. Therefore, overexpression of non-caveolar Cav-1 and a lack of PTRF both contribute to enhanced lymphangiogenesis in PCa.

## MATERIALS AND METHODS

### Reagents

RPMI-1640, DMEM, foetal bovine serum (FBS), trypsin, penicillin/streptomycin solution, G418 sulfate, sodium pyruvate and glutamine were obtained from Invitrogen (Life Technologies, VIC, Australia). Non-essential amino acids (NEAA) were from Lonza (VIC, Australia). IGEPAL, leupeptin, aprotinin, 3-(4,5-dimethylthiazol-2-yl)−2,5-diphenyltetrazolium bromide (MTT), hematoxylin, rat tail collagen and nitrocellulose membranes were purchased from Sigma-Aldrich (NSW, Australia). Dimethyl sulfoxide (DMSO) was from Chem-supply (SA, Australia). Immobilon polyvinyldifluoride (PVDF) membranes were from Fisher Scientific (VIC, Australia). Random primers and Recombinant RNasin® Ribonuclease inhibitors were obtained from Promega (NSW, Australia). Rabbit polyclonal anti-caveolin-1 was from BD Biosciences (NSW, Australia), anti-PTRF from ProteinTech (NSW, Australia), anti-VEGF-A was from R&D (BioScientific, NSW, Australia), mouse monoclonal antibody to GAPDH was from Acris (Life Research, NSW, Australia).

### Cell culture

The expression of Cav-1 was experimentally altered in three human prostate adenocarcinoma cell lines, namely DU145, LNCaP and PC3. LNCaP clones expressing GFP-tagged Cav-1 (LNCaP-Cav-1-GFP) or control cells expressing GFP alone (LNCaP-GFP) were generated by transfection using transpass D2 reagent (New England Biolabs, QLD, Australia) and G418 selection. Stable down-regulation of Cav-1 expression in PC3 was previously described [33]. Two clones each of Cav-1 down-regulated cells (DU145-C1 and DU145-C5) and control shRNA-transfected cells (DU145-S1 and DU145-S2) were generated and used throughout this study.

Cells were cultured in RPMI-1640 medium supplemented with 100 i.u./ml penicillin, 100 μg/ml streptomycin, 375 μg/ml G418 sulfate and 5% (v/v) FBS (PC3) or 10% (v/v) FBS (DU145 and LNCaP cells). The cells were cultured in 5% CO2 in a humidified atmosphere at 37°C. Lymphatic endothelial cells (LECs) were previously isolated from mice expressing temperature-sensitive SV40 large T (*H-2Kb-ts*A58) [46]. LECs were propagated in DMEM medium supplemented with 10% (v/v) FBS, 100 i.u./ml penicillin, 100 μg/ml streptomycin, 1% sodium pyruvate, 1% glutamine and 1% non-essential amino-acids. LECs were maintained in 8% CO2 in a humidified atmosphere at 33°C, and transferred to 37°C, 5% CO2 for 72 h prior to experiments.

### Conditioned medium preparation

Prostate cancer cells were grown in 10 cm dishes until 70% confluent and washed twice with PBS. Cells were then incubated with 5 ml serum-free medium for 48 h. Subsequently, the conditioned medium was collected and cell debris removed by centrifugation at 400 x g for 5 min. The conditioned media were stored at −20 °C until further use.

### Western blot analysis

Prostate cancer cell lysates were prepared as described previously [34]. Equal amounts of proteins were resolved using SDS-PAGE electrophoresis and transferred to PVDF membranes. The blots were blocked with 5% (w/v) skim milk in phosphate buffer saline (PBS) for 1 h at room temperature and probed with rabbit anti-caveolin-1 (0.25 μg/ml), rabbit anti-PTRF (1.38 μg/ml) or mouse anti-GAPDH (0.1 μg/ml) antibody followed by peroxidase-conjugated donkey anti-rabbit or sheep anti-mouse secondary antibody (GE Healthcare, NSW, Australia). Subsequent visualization was conducted using SuperSignal® West Dura extended duration substrate (Thermo Fisher Scientific, VIC, Australia) and VersaDoc TM 4000 imaging system.

### Cell viability assay

The effect of prostate cancer cell conditioned medium on LEC proliferation was assessed using the MTT assay. Briefly, 1 × 104 cells per well were seeded in a 96-well plate. After overnight incubation, cells were washed twice with PBS and 100 μl of PCa cell-conditioned medium was added in triplicate wells. After 48 h of incubation, the medium was aspirated and replaced by 100 μl medium containing 0.5 mg/ml MTT. The plates were further incubated for 3 h. Subsequently, the MTT solution was aspirated, the formazan crystals were solubilized by DMSO and the absorbance at 590 nm was measured. The results are presented as percent of viability of LEC exposed to control cell conditioned media.

### Chemokinesis measurement

A wound healing assay was conducted as described previously [50]. LECs were seeded in 24 well-plates until formation of a confluent monolayer. The middle of the wells was then scraped with a 20–200 μl micropipette tip to create a wound. Cells were washed twice with PBS and placed in prostate cancer cell-conditioned medium. The width of each wound was documented promptly after wound creation (0 h) and after 6 h of incubation with the conditioned media. The wound width was measured using ImageJ software (National Institutes of Health, Bethesda, MD). The percentage of wound closure was calculated [34]. The results are presented as percent of wound closure.

### Chemotaxis assay

Directional migration was assessed using a 48-well modified Boyden chamber assay. Prostate cancer CM was added into the lower compartment of the chambers. The wells were covered by an 8 μm pore polycarbonate membrane pre-coated with rat tail collagen type 1 (100 μg/ml in 0.2N acetic acid) and equilibrated at 37°C. The upper wells were then filled with 30 × 104 cells/ml in serum free medium. After 4 h incubation, the membranes were collected, washed with PBS and the non-migrated cells were scraped from the upper face. The membranes were fixed in 4% paraformaldehyde for 30 min, stained with hematoxylin overnight and mounted using permount mounting medium. The number of migrated cells was counted microscopically [51]. Results are reported as percent of the migration of LEC towards conditioned media of control PCa cells.

### Tube formation assay

Matrigel™ (Life Technologies, VIC, Australia), a tumour-derived gelatinous protein mixture rich in growth factors and basement membrane components, able to promote blood and lymphatic endothelial cell differentiation [52, 53] was thawed at 4 °C overnight. Fifty μl of Matrigel™ were pipetted into the wells of a 96 well-plate and incubated at 37 °C for 45 min for polymerization. LECs in 100 μL of prostate cancer cell-conditioned medium were added to the pre-coated wells. After 4-6 h of incubation at 37 °C, tube-like structures were imaged and the number of branching points was counted [34, 54]. The number of branching points is presented as a percent of control cell-conditioned medium.

### Determination of VEGF-A and VEGF-C concentration in PCa cell conditioned media

VEGF-A and VEGF-C concentrations were determined using RayBio® Human ELISA kits (BioScientific, NSW, Australia) as per manufacturer's instructions. At the time of the quantification a calibration curve of VEGF-A and VEGF-C standards was prepared. The concentration of the proteins in samples was calculated using the log-log regression equation of the best fit line of the standard calibration curve. Results are presented as mean concentration of the protein.

### RNA isolation and quantitative real-time reverse transcriptase polymerase chain reaction (RT-PCR)

Total RNA was isolated using Purelink RNA mini kit® (Life Technologies, VIC, Australia) from 70% confluent prostate cancer cells. For RNA quantification, absorbance values (260, 280 nm) were measured using Thermo Fisher NanoDrop. Complementary DNA (cDNA) was synthesized from 2 μg total RNA using Omniscript RT kit (Qiagen, Victoria, Australia) in a final volume of 20 μl using random primers according to the supplier's instructions.

The cDNA was amplified using TaqMan Universal PCR Master Mix containing AmpliTaq Gold® DNA Polymerase, dNTPs with dUTP, Passive Reference 1 and optimized buffer components. The amplification was carried out through a StepOnePlus Real Time PCR System (Applied Biosystems) using universal cycling conditions (95°C, 10 min then 95°C, 15 sec; and 60°C, 1 min for 40 cycles). Gene Expression Assays (FAM™ -MGB, 20X) for human VEGF-A (Hs01052961_m1) and VEGF-C (Hs00153458_m1) were obtained from Life Technologies (VIC, Australia).

Relative quantification was done with reference to 18S ribosomal RNA (18S rRNA) and analysed using the comparative 2–ßßCT method [55]. Results are presented as relative gene expression compared to control.

### Statistical analysis

Results are reported as mean ± S.E.M. Statistical analysis was performed using GraphPad Prism (V6.0 for Windows). *p* values <0.05 were considered significant.

## CONCLUSION

This study shows that expression of non-caveolar Cav-1 by PCa cells promotes lymphangiogenesis and suggests a mechanism mediated by the panangiogenesis regulator VEGF-A. Prostate cancer lymphatic metastasis is an early hallmark of disease progression, suggesting that this novel finding could be exploited to develop novel therapeutic strategies for prostate cancer.

## SUPPLEMENTARY MATERIALS FIGURE


