# Supplementary material for: Non-caveolar caveolin-1 expression in prostate cancer cells promotes lymphangiogenesis
Source: Oncoscience. 2015 Aug 3;2(7):635–45. doi: 10.18632/oncoscience.180 (PMC4549361; doi:10.18632/oncoscience.180)
Supplement: Supplementary file 1 [file oncoscience-02-635-s001.pdf]

Non-caveolar caveolin-1 expression in prostate cancer cells promotes lymphangiogenesis

Supplementary Materials

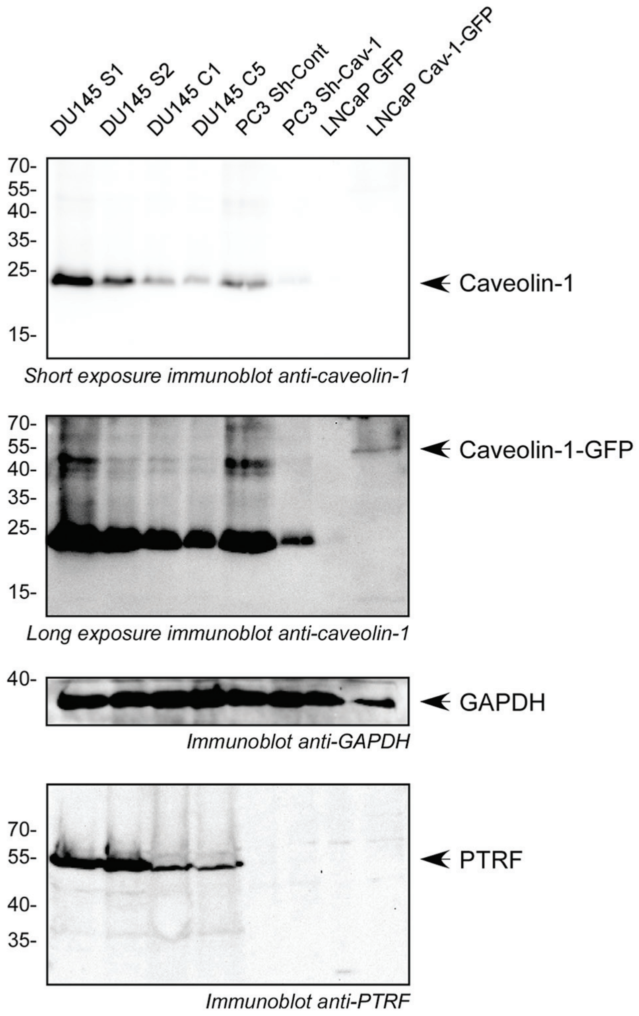

Supplementary Figure 1: Characterization of Cav-1 and PTRF expression in prostate cancer cell lines. Cell lysates from prostate cancer cells were separated by SDS-PAGE and analysed by immunobloting with anti-caveolin-1 anti-PTRF or anti GAPDH antibody as indicated. All cell lines on the same blots are shown, with caveolin-1 at long or short exposure.
